# Utility of a Phylogenetic Perspective in Structural Analysis of CYP72A Enzymes from Flowering Plants

**DOI:** 10.1371/journal.pone.0163024

**Published:** 2016-09-26

**Authors:** Wil Prall, Oliver Hendy, Leeann E. Thornton

**Affiliations:** Department of Biology, The College of New Jersey, Ewing, New Jersey, United States of America; Michigan State University, UNITED STATES

## Abstract

Plant adaptation to external pressures depends on functional diversity in cytochrome P450 (CYP) enzymes. CYPs contain structural domains necessary for the characteristic P450 fold that allows monooxygenation, but they also have great variation in substrate binding affinity. Plant genomes typically contain hundreds of CYPs that contribute to essential functions and species-specific metabolism. The CYP72A subfamily is conserved in angiosperms but its contribution to physiological functions is largely unknown. With genomic information available for many plants, a focused analysis of CYP subfamily diversity is important to understand the contributions of these enzymes to plant evolution. This study examines the extent to which independent gene duplication and evolution have contributed to structural diversification of CYP72A enzymes in different plant lineages. CYP72A genes are prevalent across angiosperms, but the number of genes within each genome varies greatly. The prevalence of CYP72As suggest that the last common ancestor of flowering plants contained a CYP72A sequence, but gene duplication and retention has varied greatly for this CYP subfamily. Sequence comparisons show that CYP72As are involved in species-specific metabolic functions in some plants while there is likely functional conservation between closely related species. Analysis of structural and functional domains within groups of CYP72As reveals clade-specific residues that contribute to functional constraints within subsets of CYP72As. This study provides a phylogenetic framework that allows comparisons of structural features within subsets of the CYP72A subfamily. We examined a large number of sequences from a broad collection of plant species to detect patterns of functional conservation across the subfamily. The evolutionary relationships between CYPs in plant genomes are an important component in understanding the evolution of biochemical diversity in plants.

## Introduction

Adaptation throughout plant evolution has been closely linked to the evolution of cytochrome P450 enzymes (CYPs). CYPs have facilitated major evolutionary advances such as cell membrane flexibility, waterproof coatings, and lignin formation [1]. Plant genomes typically have 1% of their genes encoding CYPs, which can be generally divided into four subsets: genes conserved in the plant kingdom, genes conserved in land plants, genes emerging with flowering plants, and genes contributing to specializations within plant species [2]. CYPs are critical in providing the secondary metabolism diversity that continues to facilitate plant adaptation and plant interactions with other organisms. For example, recent evidence suggests that CYP-mediated cyanogenic glucoside biosynthesis evolved multiple times for plant defense [3]. Many secondary metabolites produced by plant CYPs have important uses in human nutrition and medicine, and the tools are becoming available for engineering multi-step biosynthesis pathways for *in vitro* metabolite production [4]. Historically, plant breeding has selected for useful secondary metabolites and against harmful ones [5]. CYPs are also targets for engineering *in vitro* synthesis of novel products [6].

**Fig 1:**
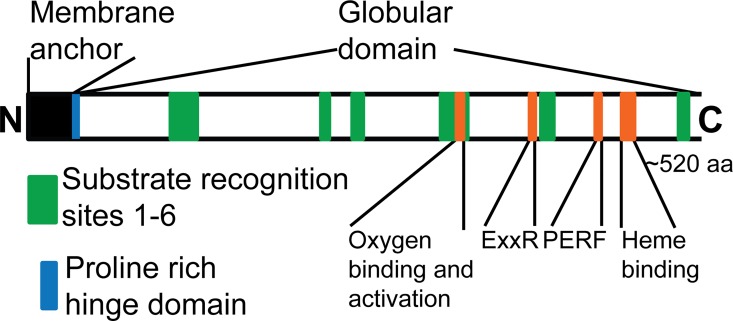
Conserved CYP domains relevant to functional analysis. Scale diagram of the domains of a CYP class II. The signature domains common in most CYPs are shown in orange. Green boxes indicate the more variable substrate recognition sites. Black and blue indicate the membrane anchor and proline rich hinge domains.

CYPs are defined as heme thiolate monooxygenases and are linked by similarity in the structural fold even though the biochemical reactions they catalyze are quite diverse. The general fold includes domains characteristic of all CYPs and those providing substrate specificity [7]. As diagrammed in Fig 1, most plant CYPs have a membrane anchor tethering them to the endoplasmic reticulum or chloroplast followed by a hinge domain. Each CYP contains domains that facilitate interactions with the heme cofactor and a cytochrome P450 reductase as well as recognition sites that facilitate interactions with various substrates. The heme-binding domain is the most conserved element in the CYP enzyme family, because it provides the cysteine ligand and is rigid enough to hold the heme in place for oxygenation [8]. The glutamate and arginine residues in the ExxR domain and the arginine in the PERF domain are conserved in all plant CYPs and appear to lock the heme pocket into place and stabilize the core structure [9]. The meander region in which the PERF domain lies is also important for protein-protein interactions with the cytochrome P450 reductase [10]. The I-helix contains conserved residues that form a proton groove critical for cleaving the O-O bond to generate the active Fe-O hydroxylating species [8]. The substrate recognition sites (SRSs) are hypervariable regions that have a conserved location in the CYP fold, but vary in amino acid functionality to accommodate diverse substrate sizes and chemistries [11].

CYPs are typically named and organized based on amino acid sequence similarity and gene structure. CYPs are organized into families that are 40% identical and subfamilies that are at least 55% identical with corresponding numbers and letters to show groupings (e.g. CYP72A is a subfamily of the CYP72 family) [12]. There are some exceptions in the plant CYPome that are resolved with additional phylogenetic analysis. Intron position and phase is often used along with amino acid sequence similarity to further examine phylogenetic relationships among CYPs [9].

In plants, the CYP72 clan is one of the largest groups of enzymes contributing to secondary metabolism for which little biochemical information is known. Clans are clusters of CYP families showing relationships between the families that predate major evolutionary divisions, such as the separation of gymnosperms and angiosperms [13]. While the CYP72 clan appears to predate land plants, most of the families and functions within the clan are divergent between algae and land plants [14]. Within the CYP72 clan, the CYP72A subfamily has been recovered in each eudicot that has been sequenced, but the biochemical capabilities in secondary metabolism appear to be quite varied. In one instance, two CYP72A enzymes evolved to catalyze subsequent steps in the same biochemical pathway. CYP72A1 from *Catharanthus roseus* is a secaloganin synthase and CYP72A224 is involved in an earlier step in the same pathway [15,16]. However, it appears to be more typical that closely related CYP72As are involved in different pathways, suggesting that CYP72A functional evolution is independent of the other CYP72As in each plant species. *Oryza sativa* CYP72A31 is involved in inactivating an acetolactate synthase-inhibiting herbicide, but the closest homologs, CYP72A32 and CYP72A33, provide no herbicide tolerance [17]. Even though herbicide inactivation is a new trait, this study suggests that CYP72A31 can inactivate foreign compounds that are not similarly modified by the close homologs. Several CYP72As from the plant order Fabales have been implicated in triterpenoid biosynthesis, but there is no evidence of an evolutionary connection between the enzymes recruited for triterpene synthesis (reviewed in [18]). Triterpene synthesis appears to have evolved multiple times and the CYP72As are among the enzymes independently recruited to this chemical pathway [19]. Other CYP72As have been implicated in herbicide or fungicide tolerance through gene expression, but their direct biochemical contributions to plant metabolism are unclear [20,21]. Within the CYP72 clan, the CYP72A sequences are most closely related to CYP734A sequences in monocots and eudicots [13]. The CYP734A enzymes inactivate brassinosteroids for plant growth regulation, but the CYP72A enzymes show no biochemical connection to steroid hormone modification.

A robust analysis of the phylogenetic relationships within the CYP72A subfamily is needed to understand functional evolution within a plant species and between closely related species. There have been studies examining the entire CYPome in a few species that included a comparison of the CYP72A subfamilies. For example, Nelson and colleagues compared the CYPomes of *O*. *sativa* and *A*. *thaliana* to determine that the major CYP families existed prior to the monocot/dicot divergence [13]. When only a few sequences are sampled for a subfamily, it is not clear whether there are independent duplications in each species or whether there is orthologous conservation of sequences between species. A broad phylogenetic analysis of CYP subfamily members from many species allows a more detailed picture of the evolutionary history that can be used to raise functional hypotheses [22].

To better understand the contributions of the CYP72A enzymes to plant metabolism, we set out to determine key structural features in the CYP72As and potential limitations on biochemical activities within the subfamily. We are testing the hypothesis that closely related CYP72A sequences will reveal functional amino acid differences that have evolved in each lineage. We surveyed land plants for CYP72A sequences and performed a phylogenetic analysis to determine evolutionary relationships within the CYP72A subfamily. Our analysis of 209 sequences from one CYP subfamily offers an important perspective on the contributions of these enzymes across angiosperms. The CYP72A phylogeny was used to guide analysis of the structural features that are conserved in more similar CYP72A sequences. We show that there are amino acid changes within important structural domains that could allow functional differences between subsets of CYP72A sequences.

## Results

### CYP72A annotation and alignment

Our search criteria required 55% sequence identity and maintenance of the structural and functional domains (Fig 1), which recovered only angiosperm sequences. The closest gymnosperm sequences in our search were ~40% similar to the angiosperm CYP72As, and there were no closely related bryophyte or fern sequences. Therefore, the set of sequences analyzed was restricted to angiosperms. The CYP734A subfamily is found in most plants and serves as the closest relative to the 72A subfamily, so the CYP734A1 sequence from *A*. *thaliana* was used to root the phylogenetic tree.

Several rounds of alignment were done to cull sequences that disrupted the general alignment trends for the CYP72A subfamily. The final alignment used for phylogenetic analysis is available in FASTA format as S6 Fig. The N-terminal domain of all the sequences was truncated prior to the first conserved proline to compare the globular/functional domain and avoid possible bias in the phylogeny from the highly divergent membrane binding domains (S1 Fig). Sequences with poor alignment (i.e. <55% amino acid identity to other CYP72As, and the presence of large or small gaps within the trimmed alignment), partial or extended sequences (i.e. sequences with less or significantly greater than 567 amino acids or obvious mistakes in intron/exon annotation), incomplete P450 sequences (i.e. alignments without P450 conserved regions: EXXR, PERF, Oxygen/heme binding, SRS1-6), and identical sequences with variable names were removed. The final CYP72A family phylogenetic reconstruction was based on 209 truncated sequences representing 33 different species from 12 orders (Table 1). This collection of sequences is a broad sampling across angiosperms, but is limited by the available genome data.

**Table 1:** Taxonomic information for sequences used in the CYP72A phylogenetic analysis.

Order	Family	Genus	Species	Abbr	Sequence Count
Poales	Poaceae	*Zea*	*mays*	Zm	11
		*Oryza*	*sativa*	Os	13
		*Sorghum*	*bicolor*	Sb	6
		*Lolium*	*rigidium*	Lr	4
		*Brachypodium*	*distachyon*	Bd	13
		*Triticum*	*aestivum*	Ta	1
		*Hordeum*	*vulgare*	Hv	1
	Cyperaceae	*Echinochloa*	*phyllopogon*	Ep	2
Ranunculales	Ranunculaceae	*Coptis*	*japonica*	Cj	1
Proteales	Nelumbonaceae	*Nelumbo*	*nucifera*	Nn	8
Vitales	Vitaceae	*Vitis*	*vinifera*	Vv	8
Malpighiales	Euphorbiaceae	*Jatropha*	*curcas*	Jc	1
		*Ricinus*	*communis*	Rc	3
	Salicaceae	*Populus*	*trichocarpa*	Pt	3
Fabales	Fabaceae	*Glycyrrhiza*	*uralensis*	Gu	3
		*Glycine*	*max*	Gm	12
		*Medicago*	*truncatula*	Mt	10
		*Glycyrrhiza*	*echinata*	Ge	3
		*Cicer*	*arietinum*	Ca	6
		*Lotus*	*japonicus*	Lj	1
Rosales	Rosaceae	*Fragaria*	*vesca*	Fv	8
		*Prunus*	*persica*	Pp	4
Malvales	Malvaceae	*Theobroma*	*cacao*	Tc	1
Brassicales	Brassicaceae	*Arabidopsis*	*thaliana*	At	8
		*Capsella*	*rubella*	Cr	5
		*Brassica*	*rapa*	Br	3
	Caricaceae	*Carica*	*papaya*	Cp	6
Gentianales	Apocynaceae	*Catharanthus*	*roseus*	Cro	7
Solanales	Solanaceae	*Nicotiana*	*tabacum*	Nt	7
			*plumbaginifolia*	Np	1
		*Solanum*	*lycopersicum*	Sl	22
			*tuberosum*	St	26
Apiales	Araliaceae	*Panax*	*ginseng*	Pg	1

### CYP72A phylogenetic analysis

**Fig 2:**
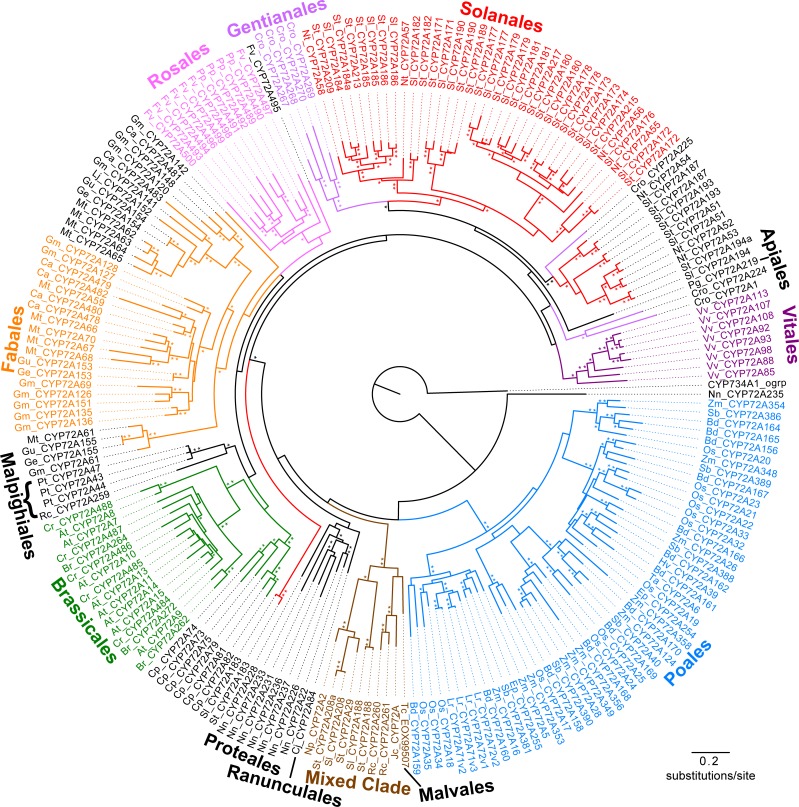
Phylogenetic tree of angiosperm CYP72A sequences. Shown is the topology from the maximum likelihood analysis of 489 aligned residues using a Jones-Taylor-Thornton substitution model. CYP734A1 from Arabidopsis was used to root the tree. A * indicates nodes with 70% or greater bootstrap support; ** indicates 95% or greater boots strap support (250 maximum likelihood pseudo-replicates). Colored lines indicate sequences from plant orders highlighted on the tree, while colored names indicated the subclades that were used for further analysis. The brown clade represents a “Mixed Clade” with sequences from three plant orders.

The CYP72A phylogeny was reconstructed using maximum likelihood, neighbor-joining and maximum parsimony criteria. The neighbor-joining tree and strict consensus tree from the maximum parsimony analysis did not have any supported conflicts when compared to the topology that resulted from the maximum likelihood analysis; here we refer to the maximum likelihood tree (Fig 2). The maximum likelihood tree was chosen because it uses a probabilistic model best fit for protein substitution; assuming different sites evolve independently and that diverged sequences evolve independently after divergence. Maximum likelihood analysis is the best method for our hypothesis that the codons for CYP72A active site amino acids have evolved independent of the codons for other amino acids within each sequence and in other sequences from the same plant. The topology shown supports a common CYP72A ancestor with the deepest nodes having high bootstrap values. The neighbor-joining and maximum parsimony trees are shown in S2 and S3 Figs.

The phylogenetic analysis of CYP72As recovered clades that are well recognized among angiosperms [23]. Monocot sequences from the Poales order are monophyletic and strongly supported with a bootstrap of 98% (Fig 2). Eudicot relationships show some exceptions to the angiosperm phylogeny. For instance, representatives of Solanales and Fabales maintain three highly supported sub-clades suggesting order-specific CYP72A expansion. The broad Solanales clade includes a subset of the *C*. *roseus* sequences, which is a species from the Gentianales order. *Vitis vinifera*, *Nelumbo nucifera*, and *Coptis japonica* sequences are excluded from the other eudicots, which is in keeping with Vitales, Proteales, and Ranunculales being ancestral to the Rosids and Asterids represented in this sequence set. Interestingly, some of the Malpighiales and Malvales sequences fall into a highly supported “Mixed Clade” that falls outside of the clade containing all of the other Rosids and Asterids. The sequences from *Vitis vinifera* grouped in a well-supported clade, suggesting the evolution of a specialized repertoire of CYP72As within this species and the possible existence of single ancestral sequence for that lineage.

**Fig 3:**
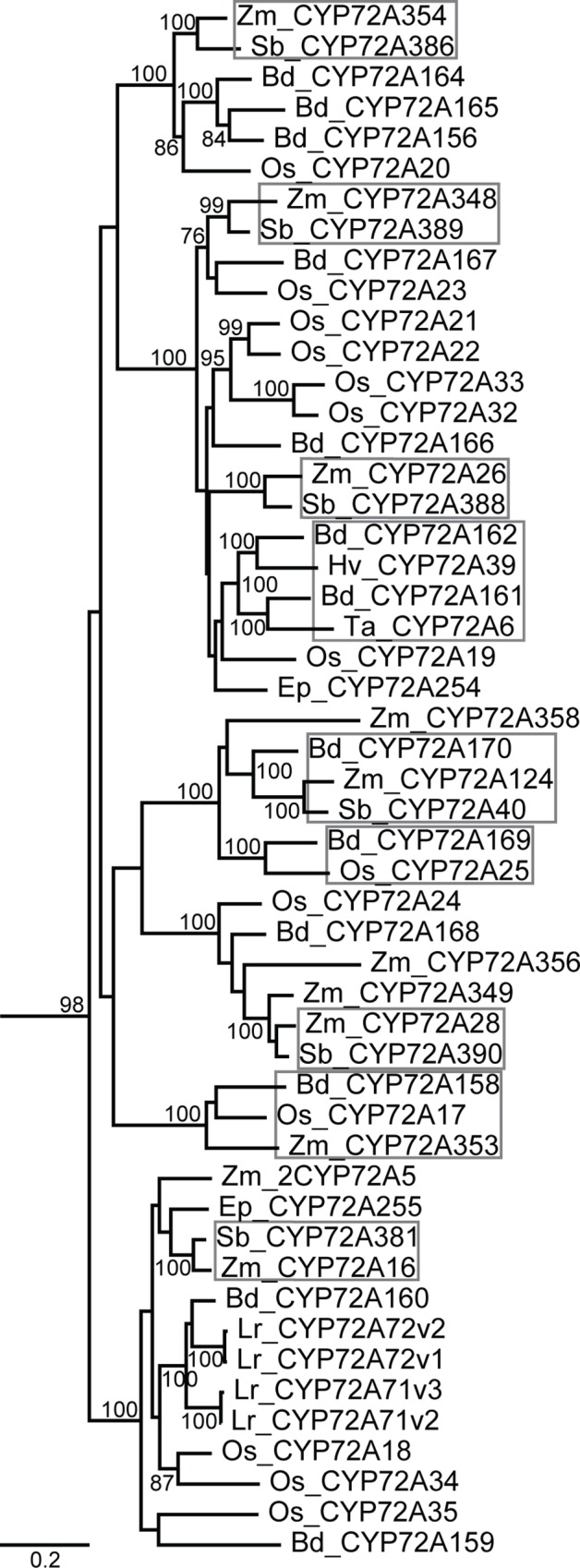
Phylogenetic relationships between Poales CYP72A sequences. Species shown include: *Zea mays* (Zm), *Oryza sativa* (Os), *Sorghum bicolor* (Sb), *Lolium rigidium* (Lr), *Brachypodium distachyon* (Bd), *Triticum aestivum* (Ta), *Hordeum vulgare* (Hv), and *Echinochloa phyllopogon* (Ep). This clade (extracted from Fig 2) is shown with bootstrap values from 250 pseudoreplicates. Orthologous groups are highlighted to show the potential for functional conservation between species.

**Fig 4:**
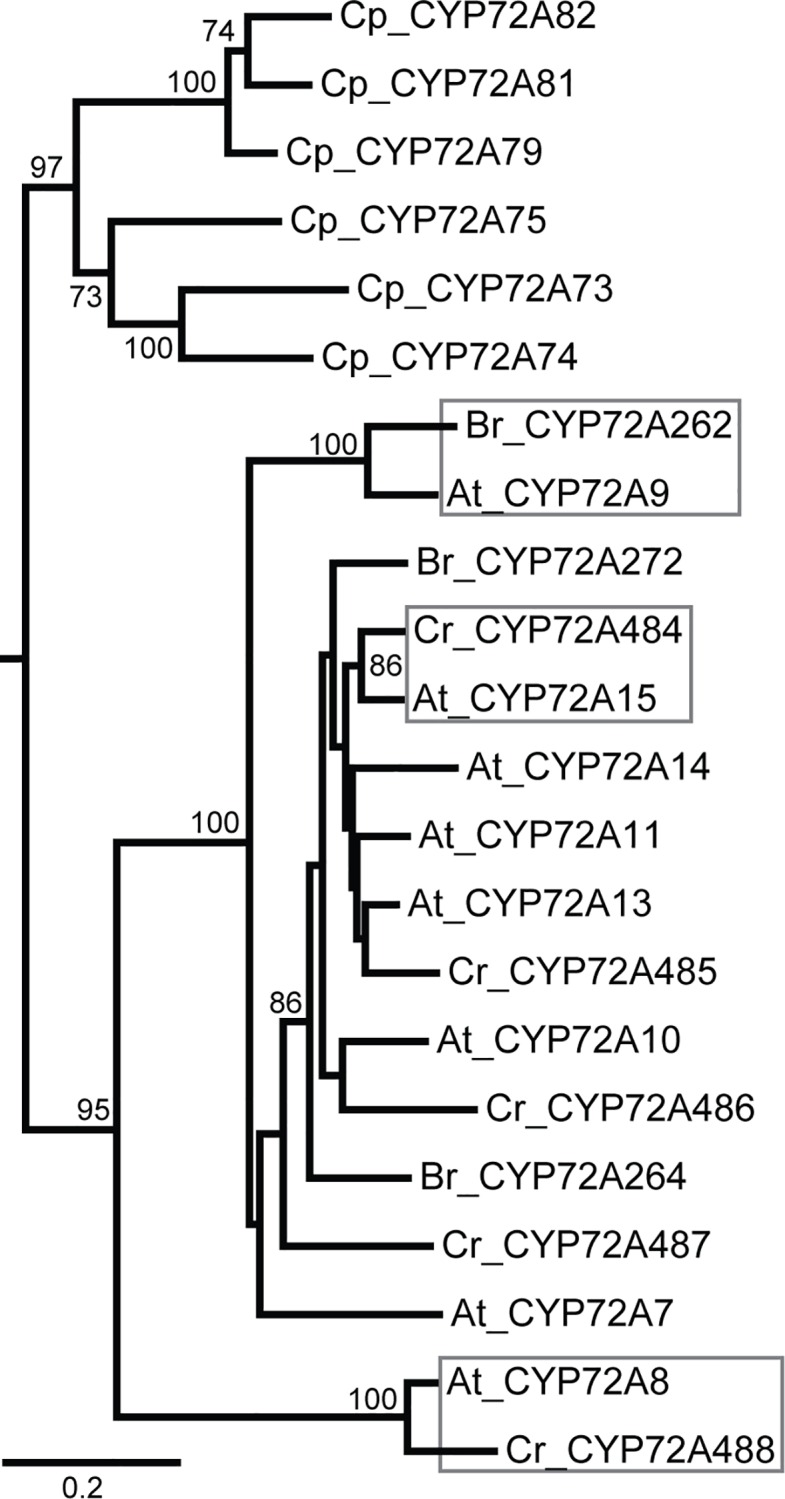
Phylogenetic relationships between Brassicales CYP72A sequences. Species shown include: *Arabidopsis thaliana* (At), *Capsella rubella* (Cr), *Brassica rapa* (Br), and *Carica papaya* (Cp). *C*. *papaya* sequences group separately from the Brassicaceae sequences within this clade. This clade (extracted from Fig 2) is shown with bootstrap values from 250 pseudoreplicates. Orthologous groups are highlighted to show the potential for functional conservation between species.

Of the Poales species that we sampled four genomes were not complete; despite that, there is some important diversity in the sequences represented. Closer examination of the structure within clades shows that Poales sequences formed two distinct groups with no specificity to species (Fig 3). All of the *Lolium rigidium* (rye grass) sequences fell into the well-supported lower portion of the Poales clade that groups separately from the majority of the Poales sequences. Since the *L*. *rigidium* genome was not completely sequenced, there could be CYP72As from this species that fall into the upper part of the clade. The topology of the Poales clade reveals well-supported sets of orthologous sequences, suggesting that the CYP72A subfamily expanded prior to speciation within these monocots. Similarly, the Brassicaceae sequences show orthologous pairings in a eudicot clade (Fig 4). Because the tips of a maximum likelihood tree are not necessarily the sequences with the highest percent identity, we verified that sequences labeled as orthologous were indeed more similar to each other than other sequences in our amino acid alignment. Orthologous pairings are also prevalent in Solanales, which is expected from the close sequence similarities between the potato and tomato genomes. Poales, Brassicales, and Solanales patterning suggests functional conservation between species within the same order and functional diversification (i.e. CYP72A expansion) prior to speciation within that order.

**Fig 5:**
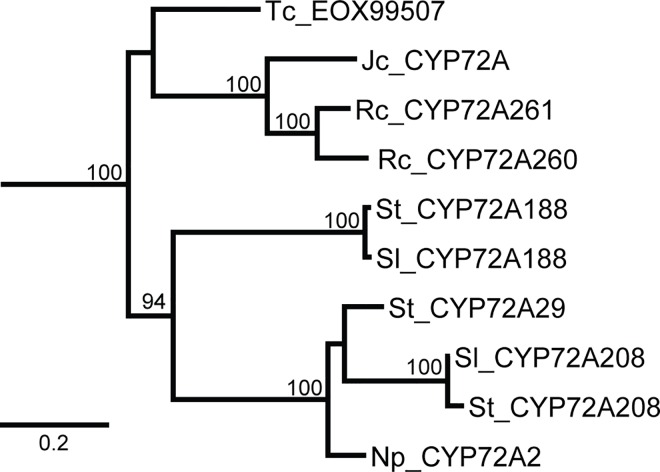
Phylogenetic relationships within the “Mixed Clade”. The mixed clade from Fig 2 shows the high level of support connecting ten sequences from six different species: *Theobroma cacao* (Tc), *Jatropha curcas* (Jc), *Ricinus communis* (Rc), *Solanum lycopersicum* (Sl), *Solanum tuberosum* (St), and *Nicotiana plumbaginifolia* (Np). This clade was included in further analysis for clade-specific structural features.

Probable gene loss is highlighted in a strongly supported eudicot clade comprised of sequences from six unrelated species, the “Mixed Clade” (Fig 5). High bootstrap support suggests a shared common ancestral gene retained in the species represented in the Mixed Clade, while the other plants most likely lost this ancestral gene. For the species *J*. *curcas*, *N*. *plumbaginifolia*, and *T*. *cacao* the only CYP72A sequences we found are grouped in the Mixed Clade. More recent annotations of the *T*. *cacao* genome suggest that there could be another CYP72A sequence that is more similar to the *P*. *persica* and *F*. *vesca* sequences than the Mixed Clade sequences (data not shown). To date, we have not found any additional complete CYP72A sequences in the *J*. *curcas* or *N*. *plumbaginifolia* genomes even though there is evidence of closely related pseudogenes.

### Amino acid variability in structural and functional domains

**Fig 6:**
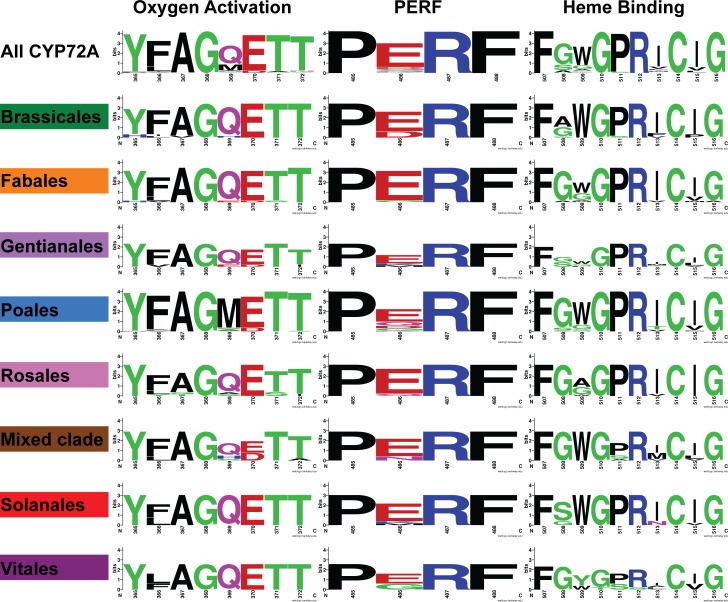
Amino acid sequence logo representations of the CYP signature domains. The positions are numbered according to the residue number in the “all CYP72A” alignment used for generating Fig 2. The relative size of the amino acid letters in the logo diagram represents the frequency of that amino acid in the subset of sequences indicated on the left. Amino acid residue frequencies from individual clades (highlighted in Fig 2) were compared to the residue frequencies across the entire set of sequences. The amino acids are colored to indicate functional similarities: (green (polar), purple (neutral), blue (basic), red (acidic), black (hydrophobic). The diagrams were created using WebLogo2 [24].

We used the relationships predicted in the maximum likelihood analysis to focus on subsets of the CYP72As for determining the frequency of particular amino acids within the SRS and structural domains. The sequences whose names are colored in Fig 2 were chosen to represent the plant order from which they came. We show distinct clade-defining differences in amino acid composition within the highly conserved functional domains (Fig 6). For the Poales clade, oxygen binding position 369 shows a clade-defining methionine. The glutamate at position 486 of the PERF domain is less well conserved in Poales than in any of the other clades examined. Most of the Rosales sequences lack a highly conserved tryptophan at position 509 in the heme binding domain.

**Fig 7:**
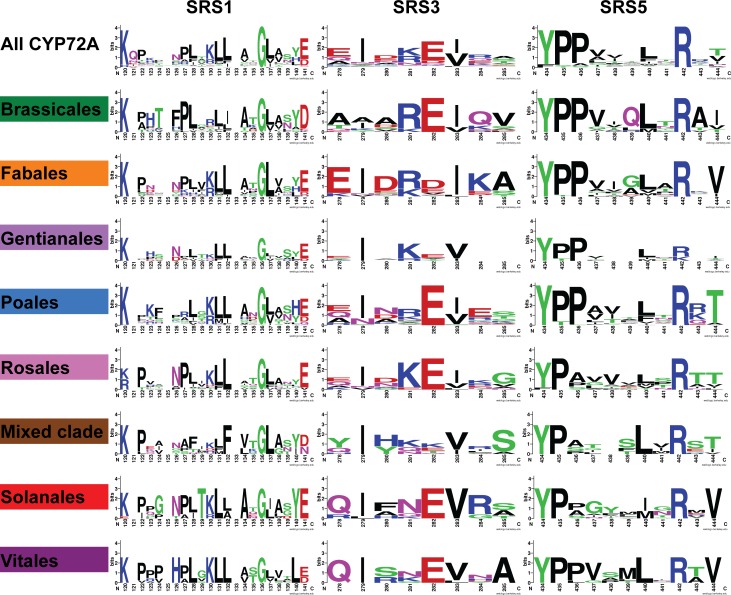
Amino acid sequence logo representations of the substrate recognition sites. Amino acid residue frequencies from individual clades were illustrated and compared as in Fig 6.

Clade-specific differences are more pronounced in the SRSs (Fig 7). Chemical properties conferred by the SRS amino acids and the shape of the substrate binding pocket determined by the R-groups that line the pocket are critical for substrate selectivity [11]. Distinct differences stand out in the Brassicales SRS regions relative to the other clades. The Brassicales SRS1 lacks a lysine at position 130, and has a more prevalent aspartate at position 141, while SRS3 shows very little conservation with the CYP72A consensus and SRS5 contains a more prevalent glutamine at position 439. The Mixed Clade also stands out for being divergent in SRS3. The Solanales clade is distinguished with a conserved threonine at position 129 in SRS1 and the Vitales clade is defined by a histidine at position 126 and a leucine at position 140 in SRS1. The Vitales leucine at position 140 is smaller than the more typical tyrosine, so it could allow a larger substrate. Similarly, SRS3 and SRS5 are missing several residues in the Gentianales group relative to the consensus, which would constrain the size of the substrate binding pocket in Gentianales CYP72As. These nonsynonymous changes in amino acids within the abovementioned domains reveal potential differences in the binding capabilities and function of the enzymes.

Some clade-defining amino acids revealed in Figs 6 and 7 cause property changes within conserved domains when compared to the CYP72A consensus. The frequency of major amino acid property changes was determined by examining the structural and functional domains from subsets of the sequence alignment based on the phylogenetic tree (Table 2.) Significant changes in amino acid properties compared with consensus reveal positions within CYP72A sequences subject to property change as well as trends shared between species and domains. For example, the Poales clade has a hydrophobic methionine in the place of the more typical glutamine in 44 of the 51 sequences examined. The Mixed Clade shows that all ten of the sequences have lost the typically negative amino acid for a positive or polar amino acid in position 280 of SRS3. A similar change occurred in all of the Vitales sequences. There are important trends that influence the potential function of the encoded enzymes in subsets of each clade. For example, changes between polar and hydrophobic amino acids in the SRS1 region for sequences from Poales would influence the shape of the binding site and the potential for stabilizing interactions with the substrate.

**Table 2:** Amino acid property changes within clades when compared to CYP72A consensus.

Functional Domain or SRS	Clade Name	Residue Position	Amino Acid Changes	Property Changes	Frequency
Oxygen Binding					
Domain	Poales	369	Q to M	Polar to Hydrophobic	44/51
PERF	Poales	486	E/D to Q/G	(-) to Polar, Hydrophobic	5/51, 5/51
	Vitales	486	E/D to G	(-) to Hydrophobic	3/8
Heme Binding					
Domain	Brassicales	508	G to A		8/16
SRS1	Brassicales	135	T/Q to K	Polar to (+)	3/16
	Fabales	123	K to N/D	(+) to Polar, (-)	7/17, 4/17
	Poales	124	P/F to N	Hydrophobic to Polar	4/51
		134	A/V to H	Hydrophobic to (+)	6/51
		135	T/Q to L	Polar to Hydrophobic	5/51
	Mixed Clade	123	K to E	(+) to (-)	4/10
SRS3	Poales	280	D/E to K	(-) to (+)	4/51
	Mixed Clade	280	D/E to H/Y	(-) to (+), Polar	4/10, 6/10
	Vitales	280	D/E to S/R	(-) to Polar, (+)	5/8, 3/8
SRS5	Poales	443	R/A to Q	(+), Hydrophobic to Polar	10/51
	Rosales	441	T/N to P	Polar to Hydrophobic	3/12

### Structural analysis of Arabidopsis thaliana CYP72A13

**Fig 8:**
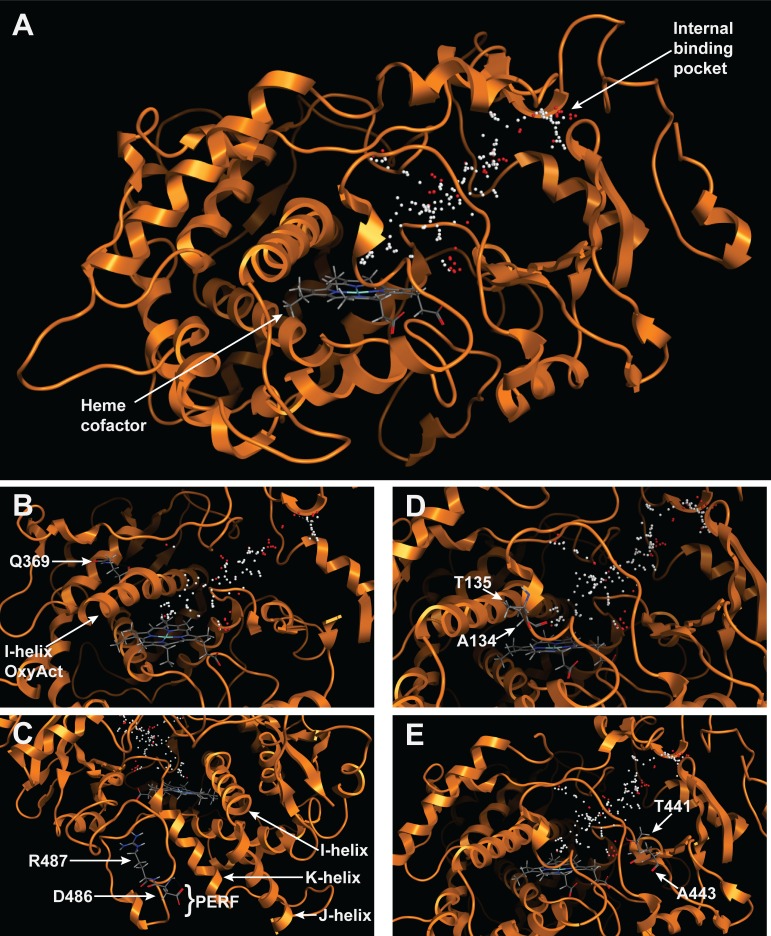
Homology model of CYP72A13 with the predicted substrate binding region highlighted with spheres above the heme cofactor. (A) Cartoon structure model showing typical CYP helices folded around the heme and an internal binding pocket. (B) Oxygen activation domain. (C) PERF domain. (D) SRS1. (E) SRS5.

Since primary sequence alone cannot reveal the extent to which an amino acid difference will impact function, it was important to visualize the position of each amino acid within the folded structure of the protein. There is no crystal structure available for a CYP72A enzyme, so homology modeling was used to predict the structure of CYP72A13 from *Arabidopsis thaliana*. CYP72A13 was chosen from the model plant, Arabidopsis, to aid in functional analysis of the subfamily in the Brassicales clade. CYP72A13 is similar to other subfamily members in *Arabidopsis thaliana* and it is orthologous to CYP72A485 from *Capsella rubella*. The CYP72A13 structure was modeled from the crystal structure of human CYP3A4, which was 24.6% identical (Fig 8A). The structural coordinates for the CYP72A13 model for viewing in a PDB viewer are provided in S7 Fig. This model had a template modeling score of 0.72 and an RMSD of 7.29Å, indicating a good global folding to the template [25]. Small spheres were placed in the predicted binding pocket to highlight the substrate binding region above the heme cofactor. Amino acid positions identified in Table 2 were located in the CYP72A13 sequence for assessing the impact of the property changes on the function of the encoded enzyme. Amino acid numbering in Fig 8 is consistent with the consensus sequence used to number the amino acids in Figs 6 and 7.

The oxygen activating domain is located in the I-helix, which runs through the center of the CYP and above the heme group. Q369 is found in the conserved I helix kink and points away from the active site (Fig 8B). This distortion in the I-helix helps to position E/D370 and T317 correctly over the heme to facilitate electron exchange during oxygenation [8]. The majority of sequences in the Poales clade have methionine instead of glutamine at position 369, which could impact the proper delivery of protons required for the typical O-O bond cleavage.

The PERF domain of CYP72A13 has the consensus D486 and R487 residues and is found on an exterior unfolded loop that runs parallel to the K-helix (Fig 8C). Importantly, this region is predicted to interact with the reductase *in vivo* and the outward projection of these residues may confer specificity within a clade or species. The PERF domain typically forms hydrogen bonds with the J-helix and K-helix to stabilize the Cys-pocket for the heme and the reductase binding site [26]. Some of the sequences from Vitales and Poales have the negative aspartate or glutamate from the PERF domain replaced with a hydrophobic amino acid, suggesting that those enzymes would have an altered shape for the reductase binding site.

In addition to the functional domains, we looked at residues with property changes in the substrate recognition sequences (Fig 8D and 8E). Residue differences in the SRS regions impact the chemical interactions between the enzyme and substrate as well as the physical constraints on substrate entry and access to the heme [11]. At SRS1, CYP72A13 has the consensus T135 and A134, which are located on the B’ helix that makes up one of the walls of the substrate binding pocket. The orientation of the B’-helix greatly impacts the local environment of the substrate binding pocket, which determines substrate selectivity [8]. Several sequences from Brassicales and Poales have property changes at these residues. Particularly for Poales, changes that influence the hydrophobicity of residues 134 and 135 could change the position of the B’-helix. Similarly, CYP72A13 has the consensus T441 and A443 residues at SRS5, which is part of the beta sheet that lines the substrate entry channel. Ten out of 51 Poales sequences have a polar glutamine in position 443, which could influence the position of the beta sheet and the size of the substrate entry channel.

**Fig 9:**
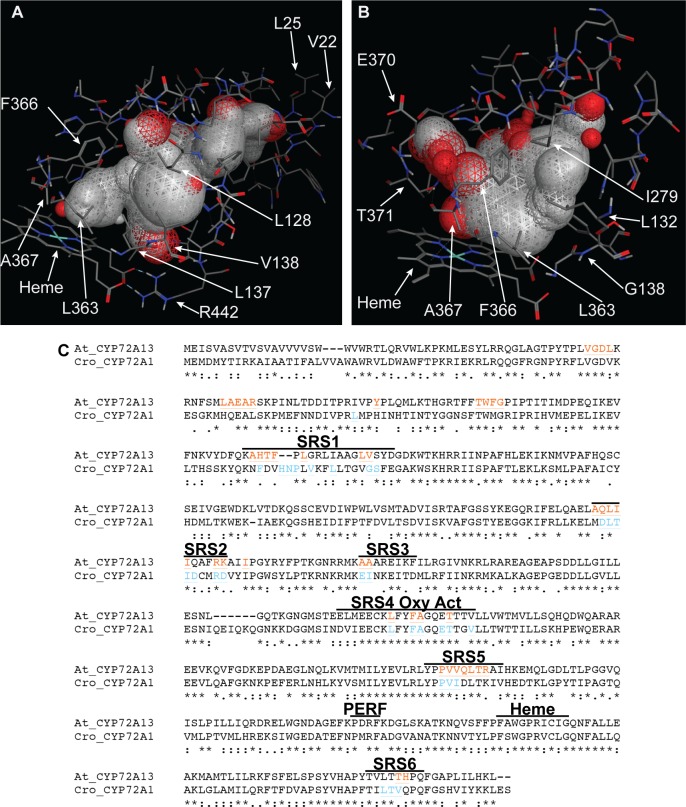
Amino acids predicted to be in the SRS regions for CYP72A13 from *Arabidopsis thaliana* and CYP72A1 from *Catharantheus roseus*. The CYP72A13 (A) and CYP72A1 (B) substrate binding sites are highlighted with space filling spheres. All amino acids within 4 Å of the spheres are shown with some amino acids labeled for reference. (C) Amino acid sequence alignment of CYP72A13 and CYP72A1 with SRS contact residues from the models highlighted in orange and blue, respectively. Predicted SRS regions and the PERF and heme-binding domains are labeled above the sequence.

In order to compare the predicted structure of the substrate binding site in different CYP72A enzymes, we modeled the structure of CYP72A1, which is a secaloganin synthase enzyme from *Catharantheus roseus*. The CYP72A1 structure was modeled from the crystal structure of human CYP3A4 (26% identical). The structural coordinates for the CYP72A1 model for viewing in a PDB viewer are provided in S8 Fig. This model had a template modeling score of 0.80 and an RMSD of 5.49Å, indicating a good global folding to the template [25]. As predicted for CYPs, the general fold of CYP72A1 was similar to CYP72A13, but the amino acids lining the substrate binding pocket differed more dramatically (Fig 9). Each model was searched for open pockets, and the region above the heme was highlighted with space-filling spheres (Fig 9A and 9B). The predicted binding pocket for CYP72A13 is relatively narrow over the heme and has a channel open to the outside of the enzyme through amino acids that are more N-terminal than the typical SRS regions (L25 and V22 are shown). CYP72A1 has a predicted binding pocket that is more spacious over the heme, and it has more room over the I-helix, which contains SRS4 (A367 and T371 are shown). The I-helix amino acids in CYP72A13 (F366 and A367 are shown) are oriented over the heme, thus preventing any substrate space above the I-helix. Fig 9C shows that the amino acids surrounding the modeled substrate binding pocket fall within the SRS regions predicted from previous publications and examined in Fig 7 [11,27]. The heme and PERF structural domains are not expected to contain substrate binding residues.

## Discussion

### Representation of CYP72As within angiosperms

The CYP72A complement for available angiosperm species ranged from 4 to 12 for the majority of species. Particular species had an expanded set of CYP72A genes while others contain only one (e.g., *Solanum tuberosum* had 26 CYP72A genes, *Theobroma cacao* had 1; Table 1). CYP72A expansion is highest in Solanales, Poales, and Fabales with *Solanum lycopersicum*, *S*. *tuberosum*, *Zea mays*, and *O*. *sativa* containing the most CYP72A sequences compared with all annotated plant genomes. At the time of our sequence collection, 19 of the 33 genomes represented had been completely sequenced. Among these species, there is a great deal of variation in the number of CYP72A sequences. For example, *Triticum aestivum* and *Lotus japonicus* have only one gene while *S*. *tuberosum* and *S*. *lycopersicum* have more than twenty CYP72A sequences. This finding suggests variation in evolutionary pressures for the types of secondary metabolites produced by the CYP72A subfamily. Expansion of CYP72As in one species relative to other species is not unique to this subfamily and has been explained in other subfamilies based on the primary function of the enzymes [28].

It is important to note that sequenced genomes primarily come from agriculturally important plants. Trait selection during the domestication process may have impacted the compliment of CYP72As differently than natural selection because secondary metabolites influence plant flavors and digestibility. Plant domestication impacts the secondary metabolite repertoire in any given species because CYPs producing undesirable chemicals are selected against and CYPs producing favorable pigments and flavors are selected for. The extent of plant domestication’s impact on our understanding of CYP72A diversity and evolution cannot be resolved until more wild relatives of crop plants have been sequenced.

### Phylogenetic relationships between CYP72As

Even though the phylogenetic tree for the CYP72A subfamily has some limitations due to lack of complete genome sequences, there are some important findings in the topology. CYP72A evolution appears to predate the divergence of monocots and dicots 200 million years ago, which was previously suggested from comparing the *O*. *sativa* and *A*. *thaliana* genomes [13]. Our more robust analysis of the CYP72A subfamily suggests that it is specific to angiosperms, because sequences were not found in an exhaustive search of gymnosperm, moss, and fern genomes. This analysis also reveals a great deal of diversity in CYP72A gene expansion across species. For example, CYP72A gene expansion is greatest in the Solanales species. It is possible that the diversity of CYP72A sequences contributed to defenses in the wild ancestor of *S*. *tuberosum* (potato) and *S*. *lycopersicum* (tomato) and domestication did not select against the presence of these enzymes. On the other hand, there are very few CYP72A sequences in *T*. *aestivum* (wheat) and *H*. *vulgare* (barley) relative to the other monocots, suggesting that domestication pressure resulted in a small number of CYP72As contributing to metabolism.

The presence of both paralogous and orthologous sequence sets in our phylogenetic tree is notable. The paralogs represent gene duplication events that happened after the speciation event separating that lineage from the last common ancestor. The duplication of individual genes, entire genomes, or chromosomal segments has been thought to be the principal source of evolutionary novelties, but most evolution models predict that one of the duplicate-gene pair will become silenced [29]. Even so, there are numerous examples of CYP families where the gene duplication event resulted in one gene maintaining the essential physiological function and others evolving new functions [30]. Here, we show that species such as *V*. *vinifera*, *N*. *nucifera*, *C*. *papaya*, *P*. *trichocarpa*, and *P*. *persica* group in species-specific clades. *C*. *roseus* does not appear to share orthologs with other species, and the secologanin synthase genes do not cluster with the other *C*. *roseus* CYP72A sequences. These findings suggest that there is a secondary metabolite repertoire unique to each distantly related species. It is possible that ancient whole genome duplication events gave rise to an ancestral set of CYP72As that is represented by the *C*. *papaya*, *V*. *vinifera*, and *N*. *nucifera* clusters on our tree. There is evidence for whole genome similarities between *C*. *papaya* and *V*. *vinifera* such as a triplicate genome structure that has been conserved in these distantly related plants because they evolve more slowly than a plant such as *A*. *thaliana* [31]. It is likely that CYP72As in plants with a more rapid life cycle have diverged more quickly, but each lineage appears to have evolved species-specific functions.

Conversely, the numerous orthologous sets in the Poales clade suggest that CYP72A functions have been conserved between grass species. For example, the *S*. *bicolor* CYP72A381 and the *Z*. *mays* CYP72A16 sequences are 93% identical and they fall into the group with the E486 to Q change in the PERF domain (Table 2). Therefore, it is possible that these enzymes would have a similar function in *S*. *bicolor* and *Z*. *mays* that is different from other CYP72As with the more typical PERF domain. This finding has important implications in further studies of secondary metabolism because functional discoveries from one plant are likely to be informative for studies of other plants. This phylogenetic tree can serve as a template for predicting functional similarities between and within species.

CYPs are critical to the balance between metabolism of important structural and signaling molecules and the metabolism dedicated to environmental response [30]. The presence of CYP72As across the angiosperms suggests that the family has played an important role in the diversity of these plants. The variation in CYP72A numbers suggests variation in the importance of the family across species. The specific contribution of each CYP72A enzyme in most plants has not yet been revealed. The relationships between CYP72As revealed in this study will help predict similarities in function between paralogs.

### Amino acid variability in structural and functional domains

The clade-specific differences we have highlighted above suggest that there is substrate binding chemistry more highly conserved within each clade. The changes highlighted in Table 2 and modeled in Fig 8 could influence substrate entry and stability as well as the efficiency of monooxygenation. Fig 9 shows that the substrate entry channel and binding pocket size are predicted to be different even when the two structures were modeled against the same template. For CYP72A13 and CYP72A1, the amino acids predicted to interact with the substrate differ more in SRS1, SRS3, and SRS5 than they do in the other highlighted domains. These results are consistent with previous associations between SRSs and variable sites for FC-X enzymes, a class of CYPs involved in xenobiotic metabolism in mammals [32]. The increase in nonsynonymous substitutions accumulating in the SRS regions in FC-X enzymes has been rationalized as a consequence of adaptive evolution, and could also explain some of the property changes we see in the CYP72A subfamily. Our phylogenetic tree was a useful guide for identifying the sequences where the most structural variation was occurring in the CYP72A subfamily. Further analysis of specific biochemical functions within each clade of the tree will show the extent of functional variation possible in the CYP72A family.

Multi-genome analysis within a CYP subfamily is important to understand the extent of CYP diversification in plants. Only 25% of the CYP genes in the well-characterized model, *A*. *thaliana*, are functionally described, and there is a tremendous amount of species-specific CYP function to be determined [2]. A focused look at a CYP subfamily across many species provided evidence of evolution within a subset of CYPs. Particularly within Poales and Solanales, the orthologous relationships will help us more quickly determine the functional contributions of CYP72As across species.

## Conclusions

The CYPomes of the fully sequenced plant genomes reflect the broad secondary metabolism differences available in each plant. The evolution of the differences in CYP sequences tracks with adaptation to many divergent external pressures, such as abiotic stresses, herbivory, pathogen attack, and pollinator preferences. This study provides a robust analysis of the CYP72A subfamily that appears in all angiosperms thus far examined. There is wide variation of numbers of CYP72As in each plant genome, which suggests differing contributions to secondary metabolism. The subfamily has blossomed in the Solanales lineage and is well represented in several grass species. We highlight major structural differences in subsets of the CYP72A subfamily that provide a useful tool in understanding some of the biochemical variation possible within groups of enzymes. This study provides a framework for deciphering CYP72A chemical contributions to plant diversity.

## Materials and Methods

### CYP72A protein sequences

A total of 209 CYP72A protein sequences were acquired via five methods: the cytochrome P450 homepage BLAST server [33], The Arabidopsis Information Resource page (TAIR; https://www.arabidopsis.org/), MaizeGDB BLAST server (http://www.maizegdb.org/), Dr. David Nelson, and through BLAST searching sequence databases (NCBI Genbank). An incomplete protein complement of CYP72A sequences had been previously identified in sacred lotus and papaya [33,34] and rice [13]. These sequences were obtained from the Cytochrome P450 Homepage. *A*. *thaliana* and *S*. *lycopersicum* sequences were used in extensive BLAST searches in Genbank to identify additional CYP72A sequences. *Z*. *mays* sequences were utilized in BLAST searches in MaizeGDB to identify the full set of CYP72A in the maize B73 genome. The CYP names used in the analysis were assigned by Dr. David Nelson; otherwise, unnamed sequences were assigned sequence tags containing the corresponding species and accession number (e.g. Tc_EOX99507). The following plant species are represented: *Zea mays* (maize), *Oryza sativa* (rice), *Sorghum bicolor* (sorghum), *Lolium rigidium* (rye grass), *Brachypodium distachyon* (purple false brome), *Triticum aestivum* (common wheat), *Hordeum vulgare* (barley), *Echinochloa phyllopogon* (late watergrass), *Coptis japonica* (gold thread), *Nelumbo nucifera* (sacred lotus), *Vitis vinifera* (grape), *Jatropha curcas* (barbados nut), *Ricinus communis* (castor bean), *Populus richocarpa* (black cottonwood), *Glycyrrhiza uralensis* (Chinese licorice), *Glycine max* (soy bean), *Medicago truncatula* (barrel clover), *Glycyrrhiza echinata* (licorice), *Cicer arietinum* (chick pea), *Lotus japonicas* (lotus), *Fragaria vesca* (strawberry), *Prunus persica* (peach), *Theobroma cacao* (cocoa tree), *Arabidopsis thaliana*, *Capsella rubella* (red shepherd’s purse), *Brassica rapa* (oil Seed), *Carica papaya* (papaya), *Catharanthus roseus* (Madagascar periwinkle), *Nicotiana tabacum* (tobacco), *Nicotiana plumbaginifolia* (tex mex tobacco), *Solanum lycopersicum* (tomato), *Solanum tuberosum* (potato), and *Panax ginseng* (ginseng) (Table 1). To be included in the set, sequences had to be >55% identical and appear to be entire. Sequences with large gaps (particularly in important structural motifs) or insertions relative to the entire set were excluded. In order to root the CYP72A phylogenetic tree, *A*. *thaliana* sequence CYP734A1 was chosen as an outlier based on previous phylogenies [12].

### CYP72A gene alignment

All retrieved CYP72A amino acid sequences from 33 species were aligned with CYP734A1 using MUSCLE [35], following similar procedures as previous studies [36]. Manual adjustments to the alignment were performed using Mesquite 2.75 [37].

### Phylogenetic analysis

Maximum likelihood analysis was performed using Garli 2.01 [38], under the Jones, Taylor, Thornton substitution model [39]. Data was analyzed using five replicates; each replicate was iterated five times to obtain the most relevant tree. The topology associated with the best tree was selected based on the likelihood score. Bootstrap analysis was performed under the same model, 250 maximum likelihood pseudoreplicates were used for two generations. All trees were visualized using FigTree 3.1 [40].

Maximum parsimony and neighbor-joining analysis was performed using *MEGA* version 6 [41]. In both analyses, 386 aligned residues were used. For maximum parsimony, data was analyzed using the tree-bisection-regrafting algorithm starting with ten initial trees and search level one with strict consensus. Bootstrap analysis was performed with 500 replicates. For neighbor-joining, the Jones, Taylor, Thornton amino acid substitution model was used and bootstrap analysis was performed with 500 replicates. These trees were compared to the maximum likelihood tree for branch consistency.

### Measuring amino acid variability in SRS regions

WebLogo3 was used to quantitatively measure the relative amounts of site specific variability within the structural domains and SRS regions [24]. Sequence variability measured in bits, 40 stacks per line, stack widths scaled, color scheme indicative of neutral, polar, acidic, basic, and hydrophobic residues as defined by WebLogo3, range chosen based on figure. The SRS regions selected for this analysis were based on the regions contributing to the active site of other CYP72A enzymes [11,27].

### Homology modeling of CYP72A13

Molecular Operating Environment (MOE, Chemical Computing Group, Montreal, Canada) was used for sequence alignment and homology modeling of CYP72A13. As the membrane binding domain in the N-terminus is absent in crystal structures of CYPs, the first 33 amino acids of the CYP72A13 primary sequence were removed prior to alignment and modeling. A homology search through the Protein Data Bank (PDB; http://www.rcsb.org; [42]) identified 1TQN (CYP3A4, Homo sapiens, [43]) as having the greatest sequence similarity to CYP72A13 (24.6%).

CYP72A13 was modeled against the CYP3A4 template, with the heme group selected as an override for the template. Settings included Amber12:EHT as the forcefield and the dielectric constant set at 1. A total of 30 models were created, and the heme group from the CYP3A4 crystal structure was manually inserted to the structure and manually bound to the conserved cysteine residue. The structure preparation feature in MOE was used to correct charges and adjust hydrogen placements. Energy minimization was performed using the Amber12:EHT forcefield parameter with a threshold of 0.1 RMS kcal/mol/Å2. The models with the lowest potential energy, Ramachandran outliers, and atom clashes were used for analysis. Energetically favorable sites within the molecule were found using Site Finder in MOE, and the region found above the heme was highlighted with positional spheres; red spheres indicate low energy positions for charged atoms and white indicated uncharged atom positions.

The primary sequence of CYP72A1 from *Catharanthus roseus* was modeled on the I-TAASER homology modeling server to the human CYP3A4 template [44–47]. The membrane binding region was identified based on an alignment to CYP3A4, and the first 27 amino acids were removed. The template had sequence identity of 26% to CYP72A1. The heme ligand from CYP3A4 was attached to the corresponding cysteine in the CYP72A1 model and energy minimized with the AMBER12 forcefield. The predicted binding site was found using the site finder feature in MOE, and residues within 4Å of the site were identified.

## Supporting Information

S1 Fig Membrane anchor sequence alignment for CYP72As. The membrane binding domain was removed so that all sequences began with the conserved proline amino acid in the hinge domain. This membrane domain is more conserved within species than between species.
(PDF)

S2 Fig Neighbor-joining tree of angiosperm CYP72A sequences. Shown is the topology from the neighbor-joining analysis of 386 amino acids using the Jones-Taylor-Thornton substitution model for calculating evolutionary distance. The tree is rooted on Arabidopsis CYP734A1. Bootstrap values from 500 replicates that are at or above 0.70 are shown.
(PDF)

S3 Fig Maximum Parsimony tree of angiosperm CYP72A sequences. The most parsimonious tree is shown from the maximum parsimony analysis of 386 amino acids using the tree-bisection-regrafting algorithm with search level one. The tree is rooted with the Arabidopsis CYP734A1 sequence. Bootstrap values from 500 replicates are shown when they are at or above the 70% cutoff.
(PDF)

S4 Fig FASTA sequence file of CYPs used in this study. (TXT)

S5 Fig Table of gene names and accession numbers for CYPs used in this study. (XLSX)

S6 Fig FASTA sequence file of aligned CYPs used in the phylogenetic analysis. (TXT)

S7 Fig Structural coordinates for the CYP72A13 model in PDB file format. This file can be read by a PDB viewer.
(TXT)

S8 Fig Structural coordinates for the CYP72A1 model in PDB file format. This file can be read by a PDB viewer.
(TXT)
