# Supplementary figures and images for: Utility of a Phylogenetic Perspective in Structural Analysis of CYP72A Enzymes from Flowering Plants
Source: PLoS One. 2016 Sep 26;11(9):e0163024. doi: 10.1371/journal.pone.0163024 (PMC5036807; doi:10.1371/journal.pone.0163024)

Supplemental Fig S2. Neighbor-joining tree of angiosperm CYP72A sequences.

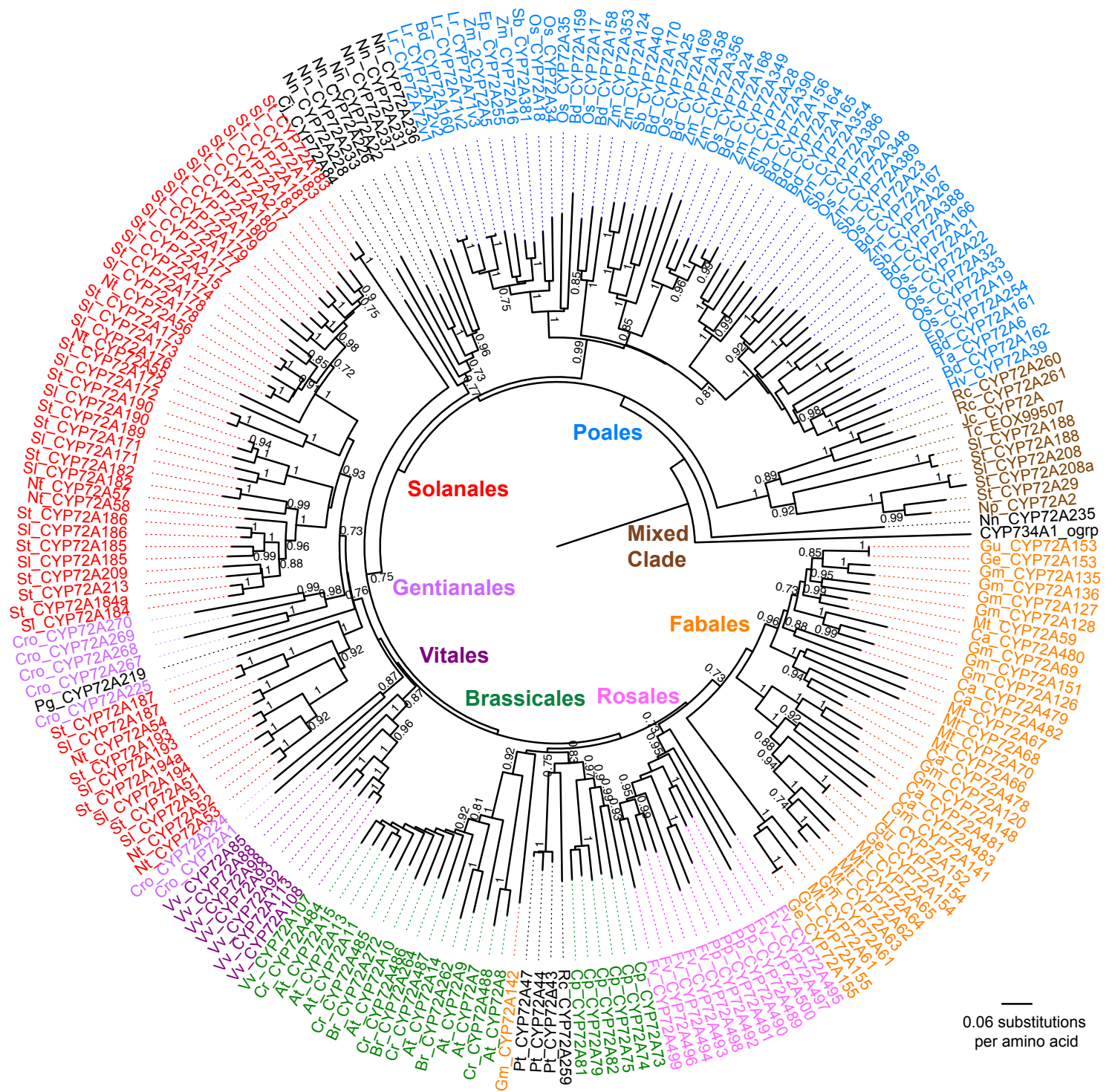

Supplement: S2 Fig — Shown is the topology from the neighbor-joining analysis of 386 amino acids using the Jones-Taylor-Thornton substitution model for calculating evolutionary distance. The tree is rooted on Arabidopsis CYP734A1. Bootstrap values from 500 replicates that are at or above 0.70 are shown. (PDF) [file pone.0163024.s002.pdf]

Supplemental Fig S3. Maximum Parsimony tree of angiosperm CYP72A sequences.

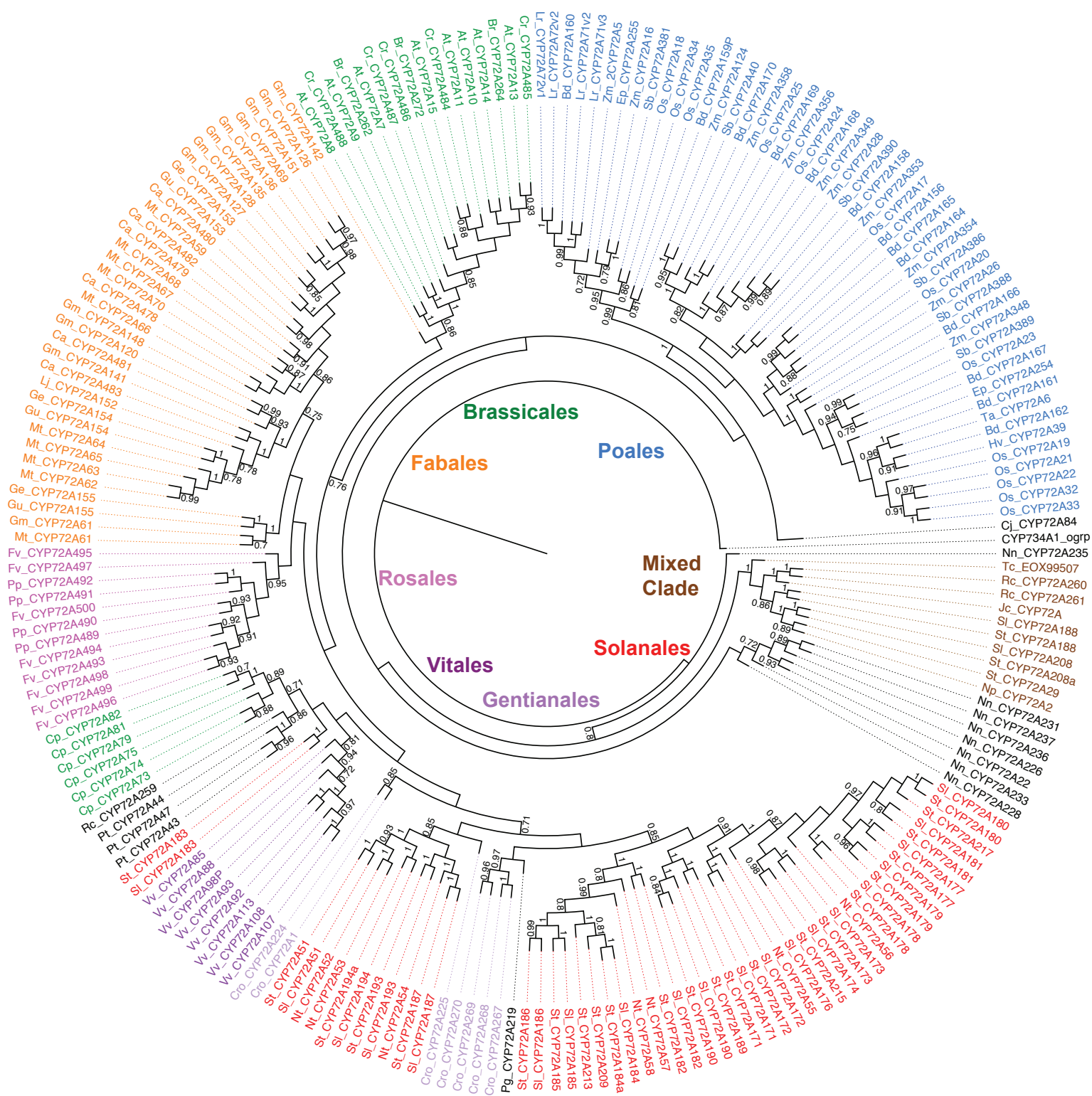

Supplement: S3 Fig — The most parsimonious tree is shown from the maximum parsimony analysis of 386 amino acids using the tree-bisection-regrafting algorithm with search level one. The tree is rooted with the Arabidopsis CYP734A1 sequence. Bootstrap values from 500 replicates are shown when they are at or above the 70% cutoff. (PDF) [file pone.0163024.s003.pdf]
