# Supplementary material for: Utility of a Phylogenetic Perspective in Structural Analysis of CYP72A Enzymes from Flowering Plants
Source: PLoS One. 2016 Sep 26;11(9):e0163024. doi: 10.1371/journal.pone.0163024 (PMC5036807; doi:10.1371/journal.pone.0163024)

Supplemental Fig S1: Membrane anchor sequence alignment.

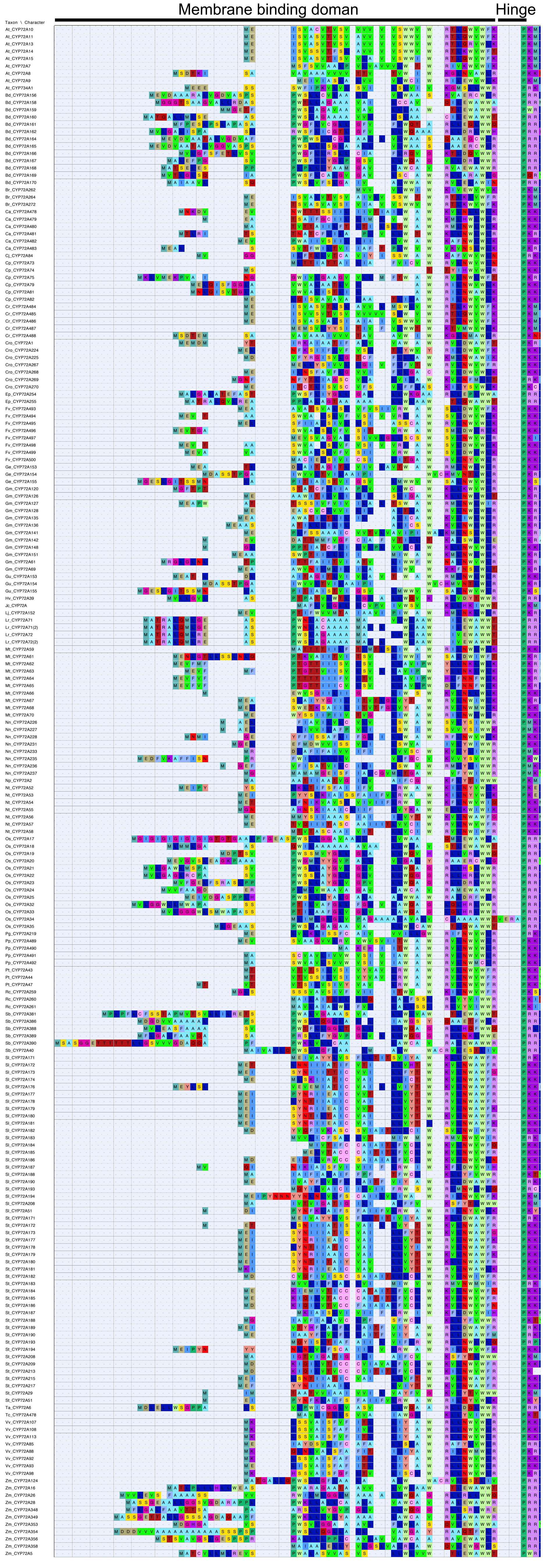

Supplement: S1 Fig — The membrane binding domain was removed so that all sequences began with the conserved proline amino acid in the hinge domain. This membrane domain is more conserved within species than between species. (PDF) [file pone.0163024.s001.pdf]
